# Imaging of Medical Patients with Acute Kidney Injury: Patterns of Ultrasound Use and the Role of Point-of-care Ultrasound at a Tertiary Care Center

**DOI:** 10.1007/s11606-025-09704-2

**Published:** 2025-07-09

**Authors:** Mathilde Gaudreau-Simard, Sydney Ruller, Melissa Dann, Michael Y. Woo, Ranjeeta Mallick, Matthew D. F. Mcinnes, Edward G. Clark, Jessica Evans

**Affiliations:** 1 Division of General Internal Medicine, Department of Medicine, The Ottawa Hospital, 1053 Carling Ave, Ottawa, ON Canada; 2 Clinical Epidemiology Program, Ottawa Hospital Research Institute, Ottawa, ON Canada; 3 Department of Medicine, University of Ottawa, Ottawa, ON Canada; 4 Department of Emergency Medicine, The Ottawa Hospital, Ottawa, ON Canada; 5 Ottawa Methods Center, Ottawa Hospital Research Institute, Ottawa, ON Canada; 6 Department of Diagnostic Imaging, The Ottawa Hospital, Ottawa, ON Canada; 7 Division of Nephrology, Department of Medicine, The Ottawa Hospital, Ottawa, ON Canada

**Keywords:** acute kidney injury, POCUS, ultrasound

## Abstract

**Background:**

The etiology of acute kidney injury (AKI) can be divided into pre-renal, renal, and post-renal causes. Ultrasound is the test of choice to identify post-renal AKI. While ultrasound is routinely used in the assessment of AKI, obstructive AKI is rare, leading to concerns of potential test overutilization.

**Objective:**

Our primary aim is to describe patterns of use of imaging in patients admitted to hospital with AKI and to determine whether imaging patterns reflect risk of obstruction. Our secondary aim is to identify the role of point-of-care ultrasound (POCUS) when assessing patients with AKI.

**Design:**

This is a retrospective cohort study.

**Participants:**

Patients admitted to internal medicine with AKI over a 12-month period at a large tertiary care academic center.

**Main Measures:**

Our outcome variables were radiology-performed ultrasound, computed tomography (CT), or point-of-care ultrasound (POCUS), presence or absence of hydronephrosis and urological intervention.

**Key Results:**

The proportion of patients with imaging was highest among those with a high-risk score and lowest in patients with a low-risk score (66.0% versus 52.2%). For radiology ultrasound specifically, the rate was 19.5% in low-risk patients and 17.7% in high-risk patients. The prevalence of hydronephrosis among patients at low, moderate and high risk for hydronephrosis was 7.1%, 8.5% and 19.7%, respectively and the rate of urological intervention was 1.4%, 1.2% and 3.8%, respectively. In moderate to high-risk patients, POCUS had a sensitivity of 86.7% and specificity of 90.0% for the identification of hydronephrosis.

**Conclusions:**

In our cohort, nearly 20% of radiology ultrasounds are ordered in patients with a low risk of obstructive uropathy, despite low rates of hydronephrosis and hydronephrosis requiring intervention in this group. With a sensitivity of 86.7% and specificity of 90.0% in patients at moderate to high risk of obstruction, POCUS may support clinical decision making in patients with AKI.

## INTRODUCTION

Acute kidney injury (AKI) affects 1 in 5 adults worldwide^1^ and is associated with a two-fold increased risk of mortality amongst patients admitted to hospital.^2^ The etiology of AKI can be divided into pre-renal, renal, and post-renal obstructive causes, with ultrasound being the imaging of choice to identify hydronephrosis, a cardinal sign of post-renal obstructive AKI.^3–6^ While ultrasound is routinely used in the assessment of AKI, obstructive AKI and the sonographic identification of hydronephrosis is rare,^5,7^ leading to concerns of potential test overutilization.^8,9^

To support a targeted approach in the use of ultrasound in AKI, studies have identified risk factors associated with obstructive uropathy to better predict those who would benefit most from imaging.^7,10–12^ Licurse and colleagues developed a risk stratification score that combines factors associated with increased risk of obstructive uropathy, such as a history of urinary obstruction, as well as factors that decrease the likelihood of urinary obstruction, such as heart failure or sepsis.^10^ Patients with a low risk of hydronephrosis according to this tool had low rates of hydronephrosis requiring intervention, suggesting that ultrasound is low yield in these patients.^10,11^ Moreover, in 2021, the American College of Radiology released appropriate use criteria (AUC) for imaging in renal failure which aligns with the work done by Licurse and colleagues.^5^ Citing the low incidence of hydronephrosis among patients without risk factors for obstructive uropathy, they highlight that the use of ultrasound in AKI is highest yield among those with risk factors for urinary obstruction.^5^ Despite the evolving evidence and recommendations to restrict imaging in the evaluation of AKI to patients with risk factors for obstructive uropathy, it is unclear whether clinicians have adopted this targeted approach.

Beyond the risk-based approach recommended in the 2021 AUC for imaging in renal failure, the use of Point-of-Care Ultrasound (PoCUS) to assess for hydronephrosis is increasingly supported by medical societies^13–15^ although we have previously reported that use remains limited.^16,17^ As an evolving innovation, POCUS has the potential to further refine current triaging practices for radiology-performed imaging in the evaluation of AKI, and there is a need for a deeper understanding of the potential synergistic applications of hydronephrosis risk stratification tools, POCUS, and radiological imaging in the evaluation of AKI.

Our primary aim is to describe radiological imaging patterns of patients admitted to hospital with AKI and to determine whether imaging patterns reflect their risk of obstruction. Our secondary aim is to identify the role of POCUS, in conjunction with a hydronephrosis risk assessment tool and radiological imaging, when assessing patients with AKI.

## METHODS

### Study Design

This is a retrospective cohort study of patients admitted to internal medicine with AKI between September 2022 and September 2023 at The Ottawa Hospital.

### Population

Patients admitted to internal medicine through the emergency department (ED) with a 50% increase in the sex-specific upper limit of normal for creatinine (≥ 126 umol/L for women and ≥ 150 umol/L for men) were eligible for inclusion. The Ottawa Hospital is a 1436-bed academic tertiary care centre with over 175,000 ED patient-visits per year.^18^ Historical baseline creatinine levels were identified on chart review, and patients were included if they had a 1.5 × increase in creatinine from baseline. If a prior creatinine was not available patients were included if AKI was identified as an active problem in their admission assessment. Patients were excluded if they were dialysis-dependent, kidney transplant recipients, pregnant, or palliative on admission, or if they died or transitioned to comfort care during the index admission.

### Outcome Measures

A health records review was conducted to obtain covariates of interest, including demographic information (age and sex), creatinine levels (baseline and at presentation), comorbidities (chronic kidney disease, coronary arterial disease, diabetes mellitus, heart failure, hypertension, and liver disease), risk factors for obstructive uropathy^10^ (history of abdominal or pelvic cancer or surgery, benign prostatic hypertrophy, hydronephrosis, nephrolithiasis, neurogenic bladder, recurrent urinary tract infections, urinary retention, urological cancer), home nephrotoxic medications (Canagliflozin, Candesartan, Chlorthalidone, Dapagliflozin, Empagliflozin, Enalapril, Sacubitril/Valsartan, Furosemide, Hydrochlorothiazide, Ibuprofen, Irbesartan, Indapamide, Lisinopril, Naproxen, Olmesartan, Perindopril, Ramipril, Spironolactone, Telmisartan, Valsartan, Vancomycin), and admission diagnosis. The outcome variables were whether the patient underwent radiology-performed ultrasound of the kidneys, ureter, and bladder (KUB), computed tomography (CT) of the abdomen and pelvis, or point-of-care ultrasound (POCUS) on presentation (i.e. within 48 h of presentation to hospital); and, the test, indication, date and time of imaging, and imaging results (presence or absence of hydronephrosis) were captured. All renal indications, whether renal obstruction or other renal pathology such as evaluation for nephrolithiasis, were recorded on chart review. Furthermore, we recorded whether a urology consultation was completed and whether the patient underwent urological intervention (nephrostomy tube or ureteric stent insertion). For patients who did not undergo imaging on presentation, charts were reviewed to identify whether they underwent CT or KUB later in their admission to determine whether obstruction may have been missed. If no imaging was pursued, the last creatinine of the index admission was recorded to be able to comment on AKI resolution within this group, which was defined by an improvement in creatinine to below 1.5 × the patient’s baseline.

### Statistical Analysis

The demographics were summarized using mean (standard deviation) or median (interquartile range) based on the underlying distribution for continuous variables, and frequency (%) for categorical variables (Table 1). Rates of imaging tests were presented using frequency (%) and stratified by risk category (Table 2). Risk category was computed using the Licurse score, with the exception of the race component which was not available on chart review. For determination of the Licurse score,^10^ the score component pertaining to a history of sepsis or prerenal acute kidney injury, use of pressors or hypotension was deemed positive if an admission diagnosis of sepsis or hypotension was noted. Considering race was not available for our cohort (convers 1-point in the Licurse score^10^), we modified the risk categories as follows: low risk was defined as a score of 0 to 1, moderate risk was defined as a score of 2 and high risk was defined as a score of ≥ 3. Risk categories with all original components including race are defined as ≤ 2, 3 and > 3 for low, moderate and high-risk, respectively.^10^

Rates of hydronephrosis, urological intervention, and urology consultation were presented using frequency (%) for the subgroup of patients that underwent imaging during their admission, either on presentation or later in their admission. Chi-square and Fisher’s exact tests were used to assess differences across the risk groups.

Diagnostic accuracy analysis for POCUS was performed on a subgroup of patients who underwent both POCUS and the reference standard (radiology-performed ultrasound (KUB) or computed tomography (CT) within 48 h) and who were in the medium or high-risk category. We excluded patients in the low-risk category given previous evidence showing low-yield of imaging in this group. If both CT and radiology-performed ultrasound were utilized, the reference standard was CT.

We conducted our statistical analyses using SAS 9.4 (SAS Institute INC., Cary NC, USA).

## RESULTS

**Figure 1:**
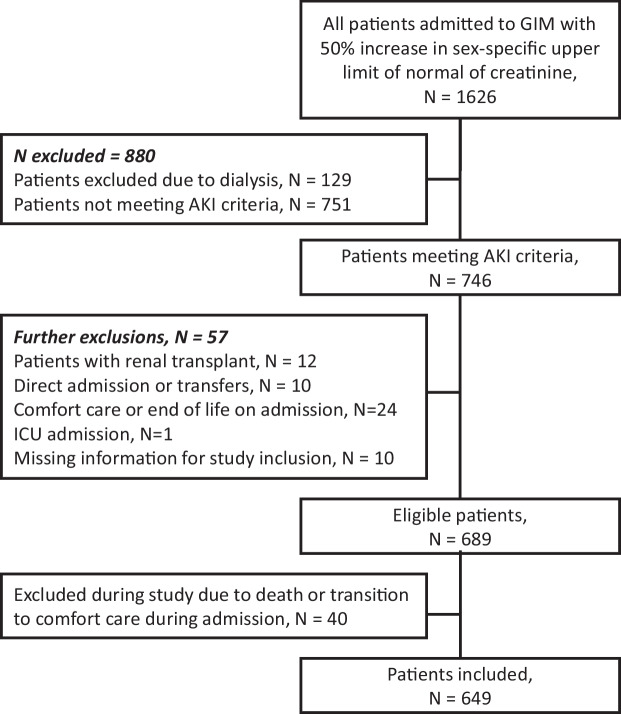
Summary of exclusion criteria.

**Figure 2:**
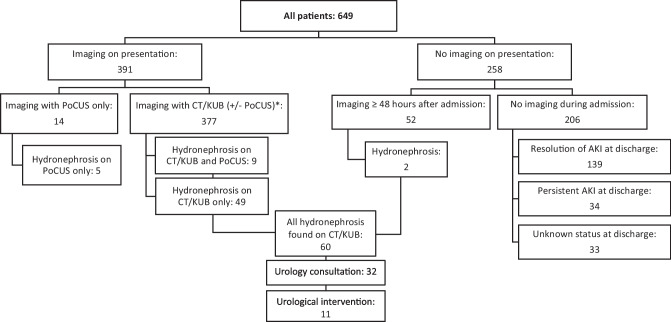
Flowchart of study outcomes. * Where POCUS was not performed, or POCUS disagreed with CT/KUB, the absence or presence of hydronephrosis was defined by the result on CT or KUB.

A total of 649 patients were included in our cohort (Fig. 1). Overall, 58.1% (n = 377) of patients underwent radiology imaging on presentation (CT or KUB) and 8.2% (n = 53) of patients underwent POCUS on presentation (Table 1). Another 8.0% (n = 52) underwent imaging over 48 h after presentation, and of the 31.7% (n = 206) of patients who did not undergo imaging during admission, 67.5% (n = 139) had resolution of AKI (Fig. 2).

**Table 1:** Cohort Characteristics of Patients who Underwent Imaging on Presentation (CT, KUB or POCUS) and Patients with no Imaging During Admission

	Total N = 649	KUB or CT n = 377	POCUS n = 53	No imaging on presentation n = 258
Mean age (SD)	72 (15)	73 (15)	72 (18)	71 (16)
Male, % (n)	51.9 (337)	54.4 (205)	62.3 (33)	47.3 (122)
Median baseline creatinine (IQR)	94 (75–121)	93 (75–120)	103 (76–122)	95 (75–121)
Median presenting creatinine (IQR)	209 (164–287)	244 (174–342)	281 (206–422)	183 (153–235)
Comorbidities and factors favoring obstruction, % (n)				
History of hydronephrosis^‡^	1.2 (8)	2.1 (8)	3.7 (2)	0
History of urinary tract infection^*^	3.9 (25)	4.5 (17)	3.8 (2)	2.7 (7)
Diagnosis consistent with possible obstruction^*§^	32.5 (211)	36.3 (137)	52.8 (28)	24.8 (64)
Comorbidities and factors supporting alternate cause, % (n)				
Heart failure^†^	21.7 (141)	19.1 (72)	15.1 (8)	26.0 (67)
Sepsis or hypotension^†^	12.3 (80)	13.3 (50)	18.9 (10)	11.2 (29)
Nephrotoxic home medications^†‖^	64.6 (419)	65.0 (245)	54.7 (29)	64.7 (167)
Other comorbidities, % (n)				
Chronic kidney disease	24.5 (159)	24.1 (91)	18.9 (10)	25.6 (66)
Diabetes Mellitus	41.1 (266)	37.9 (143)	47.2 (25)	45.6 (117)
Hypertension	63.2 (410)	61.53 (232)	62.3 (33)	65.1 (168)

ACEi – ACE inhibitor; ARB – angiotensin receptor blockers; ARNI—AKI – acute kidney injury; CT – computed tomography; IQR – interquartile range; KUB—ultrasound of the kidneys, ureter, and bladder; NSAIDS – non-steroidal anti-inflammatory; POCUS – point of care ultrasound; SD – standard deviation

^*^Presence of these elements confers 1 point in the risk score

^†^Absence of these elements confers 1 point in the risk score as they are associated with non-obstructive causes of AKI

^‡^Automatic high-risk category

^§^History of abdominal or pelvic cancer or surgery, benign prostatic hypertrophy, hydronephrosis, nephrolithiasis, neurogenic bladder, urinary retention, urological cancer

^‖^NSAIDs, Diuretics, ACEi/ARB/ARNi, Vancomycin

Compared to patients who underwent no imaging, patients who had any form of imaging on presentation (KUB, CT, or POCUS; n = 391) had a higher median creatinine on presentation as well as higher proportions of patients with comorbidities and factors favoring obstruction (Table 1). AKI was the primary admission diagnosis in 14.0% (n = 91) of patients, while other frequent diagnoses included infection or sepsis (25.0%, n = 162), as well as cardiopulmonary (13.3%, n = 86) and gastrointestinal (15.7%, n = 102) related presentations.

Forty patients were excluded from the cohort due to death or transition to comfort care during their admission (Fig. 1). Of these, 5 patients had radiology imaging on presentation (CT or KUB), with 3 patients having unilateral mild hydronephrosis. An additional 3 patients went on to undergo imaging later in their admission, with none showing hydronephrosis.

### Rates of Imaging by Risk Category

The proportion of patients with any imaging was highest among those with a high-risk score and lowest in patients with a low-risk score (66.0% versus 52.2%, p = 0.0191) (Table 2). The proportion of patients with imaging increased with increasing risk of hydronephrosis for CT (p = 0.0159) and POCUS (p = 0.0015) but remained stable or slightly decreased for KUB (p = 0.7900) across risk categories (Table 2). Noteworthy, CTs were infrequently performed specifically for a renal indication (29.8%, n = 81), but were rather conducted for other intraabdominal or a combination of indications. By comparison, KUBs were ordered for a renal indication in 96.5% (n = 110) of cases and renal POCUS was performed to assess for hydronephrosis in 100% (n = 53) of patients.

**Table 2:** Proportion of Patients by Risk of Hydronephrosis

	**Proportion of patients with imaging** % (n)			
	**Low risk of HN** ^†^ n = 113	**Medium risk of HN** ^†^ n = 248	**High risk of HN** ^†^ n = 288	
Any imaging^*^	52.2 (59)	57.3 (142)	66.0 (190)	p = 0.0191
CT	31.9 (36)	40.3 (100)	47.2 (136)	p = 0.0159
KUB	19.5 (22)	16.5 (41)	17.7 (51)	P = 0.7900
POCUS	4.4 (5)	4.8 (12)	12.5 (36)	p = 0.0015
No imaging	48.7 (55)	42.7 (106)	33.7 (97)	p = 0.0116

CT – computed tomography; HN – hydronephrosis; KUB – kidney, ureter, bladder ultrasound; POCUS – point of care ultrasound

^*^All patients with CT, KUB, or POCUS imaging within 48 h of presentation

^†^Risk of hydronephrosis was based the modified Licurse score which excluded race factor: low risk = 0–1, moderate risk = 2, high risk ≥ 3

### Outcomes by Risk Category

In the subgroup of patients that underwent imaging with KUB or CT either on presentation or later in their admission to rule out hydronephrosis (n = 443), 13.5% (n = 60) had hydronephrosis, 7.2% (n = 32) underwent urology consultation, and 12.5% (n = 11) required urological interventions (Fig. 2, Table 3). The risk of hydronephrosis increased with increasing risk category (p = 0.0017). Among low-risk patients, hydronephrosis was present in 7.1% (n = 5) and 1 patient underwent urological consultation with subsequent intervention. More specifically, the prevalence of hydronephrosis among patients at low risk who underwent KUB was 4.5% (n = 1/22). The prevalence of hydronephrosis among patients with a medium risk of hydronephrosis was 8.5% (n = 14), although over half of these patients underwent urological consultation. In comparison, the rates of hydronephrosis in the high-risk group were nearly three-fold greater compared to the low-risk group (19.7%, n = 41), with 56.1% (n = 23/41) of these patients undergoing urological consultation and 19.5% (n = 8/41) requiring an intervention (Table 3). Among patients who had delayed imaging (≥ 48 h after presentation), no patients with low or medium risk of hydronephrosis went on to be diagnosed with hydronephrosis, while 2 high-risk patients who did not undergo imaging on presentation were diagnosed with hydronephrosis later in their admission (Fig. 2).

**Table 3:** Prevalence of Urological Complications Among Subgroup of Patients who Underwent Imaging with CT and/or KUB^*^ to Rule Out Hydronephrosis

	**Prevalence of urological complication** **% (n)**		
	**HN** n = 60	**Urology consultation** n = 32	**Urological intervention**^‡^ n = 11
Low risk of HN^†^ (n = 70)	7.1 (5)	1.4 (1)	1.4 (1)
Medium risk of HN^†^ (n = 165)	8.5 (14)	4.8 (8)	1.2 (2)
High risk of HN^†^ (n = 208)	19.7 (41)	11.1 (23)	3.8 (8)
	p = 0.0017	p = 0.0088	p = 0.2417

HN – Hydronephrosis

^*^Includes results of all KUB and/or CT performed during index admission (imaging performed within 48 h of presentation as well as later during index admission)

^†^Risk of hydronephrosis was based the modified Licurse score which excluded race factor: low risk = 0–1, moderate risk = 2, high risk ≥ 3

^‡^Urological intervention defined as placement of a nephrostomy tube or ureteric stent

### POCUS as a Tool in the Assessment of AKI

The proportion of patients who underwent POCUS for those at medium and high risk of hydronephrosis was 8.9% (n = 48), which was more than twofold greater than those at low risk (4.4%, n = 5, p = 0.1588) (Table 2). For the combined group of medium and high-risk patients, POCUS had a sensitivity of 86.7% (CI 79.7–91.5) and specificity 90.0 (86.3–92.8) for the diagnosis of hydronephrosis compared to the gold standard (CT or KUB) (Table 4). We report two cases where POCUS was negative, but CT showed hydronephrosis (false negative POCUS). In both cases, hydronephrosis was graded as mild on CT, and the patient did not require urological intervention.

**Table 4:** Sensitivity and Specificity of POCUS for Presence of Radiologically Confirmed Hydronephrosis Among Patients at Moderate to High-Risk for Hydronephrosis, Percentage % (95% confidence intervals)

	**KUB or CT positive**	**KUB or CT negative**	
**POCUS positive**	13	4	PPV 76.5 (68.9–82.7)
**POCUS negative**	2	36	NPV 94.7 (91.7–96.7)
	Sensitivity 86.7 (79.7–91.5)	Specificity 90.0 (86.3–92.8)	

CT – computed tomography; KUB – ultrasound of kidneys, ureter, bladder; NPV – negative predictive value; POCUS – point of care ultrasound; PPV – positive predictive value

## DISCUSSION

We report imaging patterns in a large cohort of patients admitted to internal medicine with AKI. Whereas previous studies have focused on developing and validating risk scores for obstructive uropathy,^7,10–12^ to our knowledge, this is the first study to report on the rates of imaging, including POCUS, stratified by previously validated risk categories.

Although we observed that the rates of imaging were appropriately highest in patients at highest risk of hydronephrosis and obstructive uropathy, this was predominantly driven by increasing rates of CT with risk category, the minority of which were conducted specifically to assess for obstructive aetiologies of AKI. By comparison, radiological ultrasounds (KUB) which were ordered to assess for hydronephrosis in over 95% of cases, were performed in one fifth of low-risk patients and did not trend with increasing risk of hydronephrosis.

Previous studies have shown that the risk of hydronephrosis and urological interventions among patients at low clinical risk group is not substantial. Licurse and colleagues report a rate of hydronephrosis of 3.7% and a rate of urological intervention of 0.4%.^10^ Similarly, Ip et al. externally validated the Licurse score and reported a rate of hydronephrosis of 4.0% along with a rate of intervention of 1.1% in this low-risk group.^11^ Tummalapalli et al. also stratified patients using the Licurse score and report a rate of 4.0% of hydronephrosis in low-risk patients.^12^ Noteworthy, these studies report rates of hydronephrosis in patients with AKI who underwent KUB only (their cohorts did not include CT).^10–12^ Consistent with this prior work, we also reported low rates of hydronephrosis, of 4.5% for KUB and 7.0% for combined KUB/CT, and rare need for urological intervention (1.4%) in this group using a modified Licurse score which excluded race. Additionally, for low-risk patients who did not undergo imaging on presentation, we found no cases of hydronephrosis diagnosed on imaging later in their admission. Our study supports the existing body of evidence that dissuades the routine use of ultrasound to rule out hydronephrosis among patients with low clinical risk of urological obstruction as per a validated risk score. Considering this, the rates of KUBs conducted among low-risk patients (19.3%) in our study represent an opportunity for improved resource stewardship at our center and possibly in other large academic internal medicine settings.

Among patients at high-risk for hydronephrosis, we found that 66.2% of patients underwent imaging, with most patients undergoing CT. Although CTs were rarely performed solely to assess for kidney pathology, use of CT allowed us to track rates of hydronephrosis. In the high-risk group, we found a rate of hydronephrosis of 19.3%, comparable to Licurse and Ip's reported rates of 16.1%^10^ and 20.9%.^11^ The rate of hydronephrosis requiring intervention in our high-risk group was also similar at 3.9%, compared to the rates of 4.7%^10^ and 4.9%^11^ reported by Licurse and Ip, respectively. Our study supports that imaging is highest yield in this group as highlighted by the 2021 American College of Radiology AUC in renal failure.^5^

Our study is also the first to explore the role of POCUS as it relates to a risk assessment tool and traditional radiology imaging modalities, in a cohort of patient with AKI. The use of POCUS overall was less frequent than other imaging modalities (8.2%). Patients who underwent POCUS had a higher median presenting creatinine than those who did not, suggesting that providers perform POCUS in the most severe cases of AKI. Additionally, the rates of POCUS were over two times higher in the high-risk category than in the low and moderate-risk categories, which also suggests that clinicians are more likely to use this tool when the pre-test probably for obstruction was highest, potentially because these are the cases where rapid, bedside diagnosis is most valuable. Considering that imaging, including POCUS, has been demonstrated to be low-yield in patients at low-risk for hydronephrosis, we pursued a diagnostic accuracy analysis for patients in the moderate to high-risk categories only. We found a sensitivity of 86.7% and specificity of 90.0%, suggesting that POCUS may be a useful adjunct to risk stratification scores and may support clinical decision-making in patients with AKI who are at moderate to high risk of obstruction.

A strength of our study is the chart review design that allowed for detailed information gathering which facilitated the reliable use of the Licurse score. Limitations include the retrospective single-center nature of our study, as well as a rigid entry criteria based on creatinine cutoffs (≥ 126 umol/L for women and ≥ 150umol/L for men). Though this means that some AKIs that did not meet the entry cutoff were missed, we ensured that patients with chronic kidney disease were excluded by performing a manual review of baseline creatinine and excluding patients that did not meet the 1.5 × increase in creatinine criteria. Where baseline creatinine was not available, we supplemented with chart review to ensure AKI was identified as an issue by the clinical team, giving us the confidence that all patients in our cohort had a true AKI.

## CONCLUSION

We found that nearly 20% of KUB ultrasounds are ordered in patients with a low risk of obstructive uropathy at our institution. Using previously validated risk stratification tools, we demonstrate that the rates of hydronephrosis and hydronephrosis requiring intervention increase with increasing risk, supporting previous literature that recommends a targeted approach to imaging in patients with AKI. Finally, we reported that POCUS has a sensitivity of 86.7% and specificity of 90.0% for the identification of hydronephrosis in moderate to high-risk patients, which supports its use in clinical decision making in conjunction with risk assessment tools. Further research is needed to establish how POCUS could be used to assess patients with AKI and contribute to radiology resource conservation.

## Data Availability

Data available on reasonable request.
